# Gene silencing of endothelial von Willebrand Factor attenuates angiotensin II-induced endothelin-1 expression in porcine aortic endothelial cells

**DOI:** 10.1038/srep30048

**Published:** 2016-07-22

**Authors:** Anar Dushpanova, Silvia Agostini, Enrica Ciofini, Manuela Cabiati, Valentina Casieri, Marco Matteucci, Silvia Del Ry, Aldo Clerico, Sergio Berti, Vincenzo Lionetti

**Affiliations:** 1 Laboratory of Medical Sciences, Institute of Life Sciences, Scuola Superiore Sant’Anna, Pisa, Italy; 2 Fondazione CNR/Regione Toscana “G. Monasterio”, Pisa, Italy; 3 Institute of Clinical Physiology, National Council of Research, Pisa, Italy

## Abstract

Expression of endothelin (ET)-1 is increased in endothelial cells exposed to angiotensin II (Ang II), leading to endothelial dysfunction and cardiovascular disorders. Since von Willebrand Factor (vWF) blockade improves endothelial function in coronary patients, we hypothesized that targeting endothelial vWF with short interference RNA (siRNA) prevents Ang II-induced ET-1 upregulation. Nearly 65 ± 2% silencing of vWF in porcine aortic endothelial cells (PAOECs) was achieved with vWF-specific siRNA without affecting cell viability and growth. While showing ET-1 similar to wild type cells at rest, vWF-silenced cells did not present ET-1 upregulation during exposure to Ang II (100 nM/24 h), preserving levels of endothelial nitric oxide synthase activity similar to wild type. vWF silencing prevented AngII-induced increase in nicotinamide adenine dinucleotide phosphate (NADPH) oxidase (NOX) activity and superoxide anion (O2−) levels, known triggers of ET-1 expression. Moreover, no increase in O2− or ET-1 levels was found in silenced cells treated with AngII or NOX-agonist phorbol ester (PMA 5 nM/48 h). Finally, vWF was required for overexpression of NOX4 and NOX2 in response to AngII and PMA. In conclusion, endothelial vWF knockdown prevented Ang II-induced ET-1 upregulation through attenuation of NOX-mediated O2− production. Our findings reveal a new role of vWF in preventing of Ang II-induced endothelial dysfunction.

Endothelin (ET)-1, a potent vasoconstrictor and pro-inflammatory peptide, maintains the vascular tone in healthy humans^1^,^2^, but its expression is upregulated in various cardiovascular disorders like systemic and pulmonary hypertension^3^,^4^, myocardial infarction^5^,^6^ and fibrosis^7^. High levels of angiotensin II (Ang II), a biologically active hormone, increase ET-1 expression in endothelial cells^8^,^9^, impair endothelial nitric oxide synthase (eNOS) activity^10^ and promote endothelial oxidant stress, which together contribute to the development of endothelial dysfunction^11^.

High levels of ET-1 create a sustained and self-perpetuating loop of vascular dysfunction^12^ characterized by endothelial eNOS impairment^13^ and enhanced release of superoxide anion (O2−) following the activation of nicotinamide adenine dinucleotide phosphate (NADPH) oxidase^14^. Even if the maintenance of eNOS activity^15^ and the attenuation of O2− generation^16^ may in part counteract ET-1 detrimental effects, prevention of Ang II-induced ET-1 expression without interfering with endothelial function remains a desirable achievement. Since endothelial function directly depends on the cell phenotype^17^, we hypothesized that von Willebrand factor (vWF) may modulate ET-1 expression during Ang II stimulation, despite being so far mostly a hallmark of endothelial phenotype mainly involved in hemostasis^18^.

ET-1 increases either endothelial or circulating levels of vWF^19^,^20^ in different diseases like systemic hypertension^21^ or acute coronary syndrome^22^. Even though some studies observed that plasma vWF levels are reduced after pharmacological treatment with angiotensin-converting enzyme (ACE) inhibitors and/or angiotensin-receptor blockers (ARBs)^23^,^24^, it is still unknown whether disabling the endothelial vWF expression could prevent Ang II-induced ET-1 upregulation. It is noticeable that vWF blockade improves endothelial function in coronary patients^25^ and protects the murine heart against ischemia/reperfusion injury^26^, but the underlying mechanisms are still unknown. Unraveling how endothelial vWF prevents the onset of endothelial dysfunction could provide new avenues for protection against Ang II-induced cardiovascular injury.

In our study, we tested the effects of short interfering RNA (siRNA)-mediated gene silencing of endothelial vWF during chronic exposure to high levels of Ang II in primary porcine aortic endothelial cells (PAOECs), a well-established *in vitro* culture system for studying the molecular mechanisms underlie alterations of vWF expression^27^ and endothelial function in general^28^. Our findings reveal a hitherto unsuspected role of endothelial vWF downregulation in preventing Ang II-induced ET-1 upregulation through reduced NADPH oxidase (NOX)-mediated oxidative stress, without impairing nitric oxide (NO) production.

## Results

### Targeting vWF expression in PAOECs with siRNA

**Figure 1:**
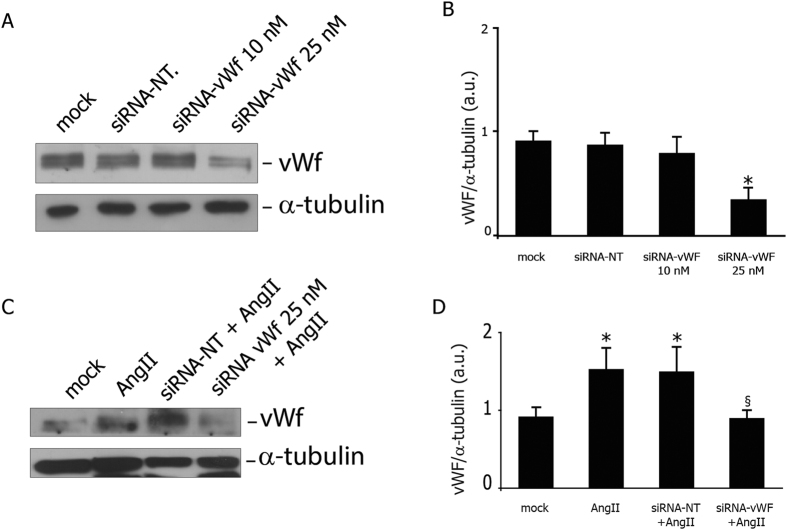
Targeting vWF expression in PAOECs with siRNA. Dose-dependent effects of cell transfection with anti-von Willebrand Factor (vWF) smart pool short interference RNA (siRNA). (**A**) representative western blot; (**B**) vWF levels are shown as arbitrary units of vWF (250 kDa, MW)/alpha-Tubulin (50 kDa, MW) ratio. (**C**) AngII-induced (100 nM for 24 h) vWF upregulation was prevented in vWF- knockdown cells. Representative western blot is shown. (**D**) Levels of vWF are expressed as arbitrary units of vWF (250 kDa, MW)/alpha-Tubulin (50 kDa, MW) ratio. Mock: mock treated cells; AngII: angiotensin II (100 nM AngII for 24 h); siRNA NT: non-targeting siRNA; siRNA vWF: anti-vWF siRNA. All measurements are mean ± SD, n = 3 independent experiments performed in triplicate. *p < 0.05 vs. mock at rest; ^§^p < 0.05 vs. AngII.

Transfection of 25 nM siRNA anti-vWF produced a significant reduction of porcine vWF protein expression in target cells (Fig. 1A,B) and the selected silencing dose was used in all of the following experiments. The reduction of vWF protein expression did not affect cell viability and growth in each experimental condition (Suppl. File: Fig. 1A).

**Figure 2:**
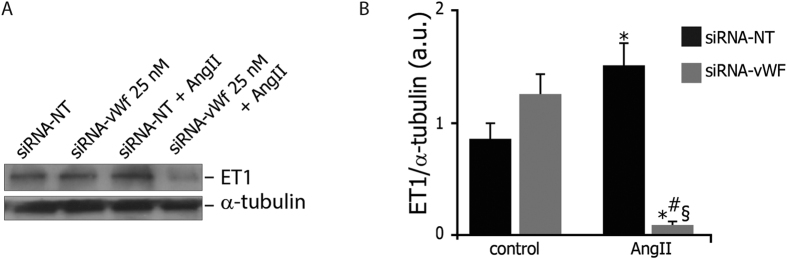
Effects of gene silencing of endothelial vWF on ET-1 protein expression. (**A**) vWF downregulation did not affect the endothelial expression of endothelian-1 (ET-1) in the presence of normal microenvironment, while strongly impairing ET-1 expression during chronic exposure to AngII. Representative western blot is shown. (**B**) Levels of ET-1 are expressed as arbitrary units of ET-1 (24 kDa, MW)/alpha-Tubulin (50 kDa, MW) ratio. siRNA NT: non-targeting siRNA; siRNA vWF: anti-vWF siRNA; AngII: angiotensin II. All measurements are mean ± SD, n = 3 independent experiments performed in triplicate. *p < 0.05 vs. siRNA-NT at rest; ^#^p < 0.05 vs. siRNA-vWF at rest; ^§^p < 0.01 vs. siRNA-NT under AngII.

RT-PCR analysis with efficiency in the range of 95–105% was performed to assess the vWF gene expression in PAOECs transfected with siRNA-vWF. vWF gene expression was markedly reduced in transfected PAOECs and the expression of the corresponding reference gene was revealed (Suppl. File: Fig. 2).

### Gene silencing of endothelial vWF prevents Ang II-induced vWF upregulation

24 h treatment with Ang II (100 nM) was used to mimic the microenvironment to which endothelial cells are exposed during cardiac dysfunction. As expected, Ang II induced an upregulation of vWF expression in wild-type cells, which was effectively prevented in vWF-knockdown cells (Fig. 1C,D). Ang II treatment and reduction of vWF protein expression did not affect cell viability and growth in each experimental condition (Suppl. File: Fig. 1B). In addition, ATR1 was similarly expressed in vWF-knockdown and wild type cells under stressed conditions (2.9 ± 1.6 vs 2.0 ± 0.6).

### Gene silencing of endothelial vWF prevents Ang II-induced ET-1 upregulation

In our culture system, we found that vWF downregulation did not significantly affect endothelial ET-1 expression in wild-type cells. Conversely, Ang II-induced ET-1 upregulation was disabled in vWF knockdown PAOECs (Fig. 2A,B).

### Gene silencing of endothelial vWF did not affect eNOS protein expression and activity

**Figure 3:**
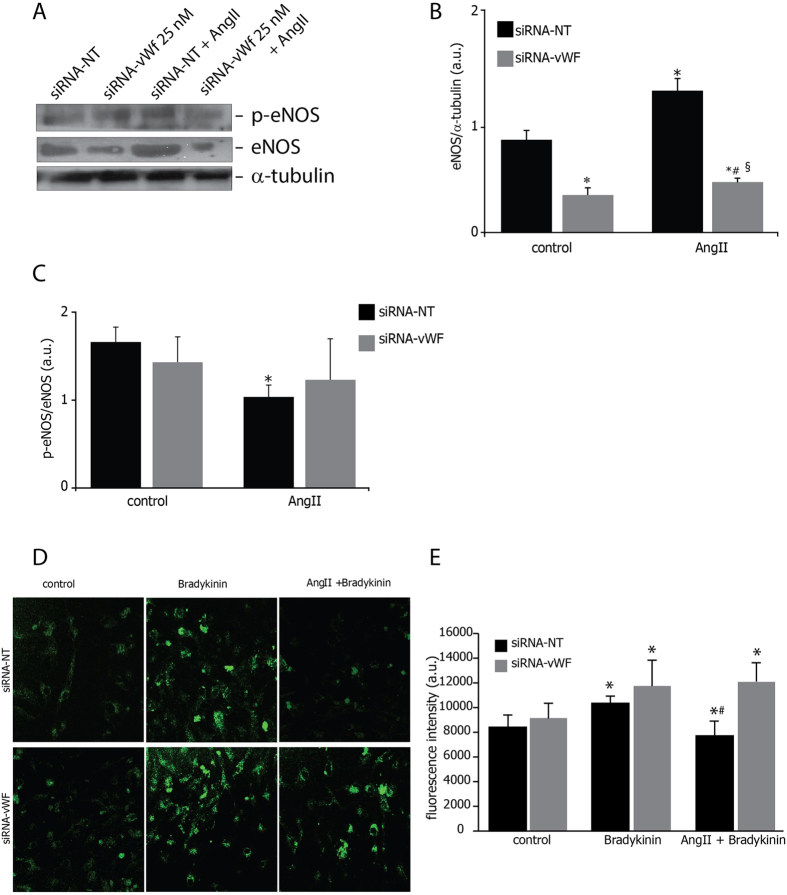
Effects of gene silencing of endothelial vWF on NO production. (**A**) vWF downregulation reduced total eNOS protein expression in resting conditions and in cells exposed to AngII. Representative western blot is shown. (**B**) total eNOS protein expression in resting conditions and in cells exposed to AngII is shown as eNOS (140 kDa, MW)/alpha-Tubulin (50 kDa, MW) ratio. (**C**) vWF downregulation did not affect the phospho-eNOS/eNOS ratio under each microenvironment. *p < 0,05 vs siRNA-NT at rest; ^#^p < 0.05 vs siRNA-vWF at rest; ^§^p < 0.05 vs siRNA-NT under AngII. (**D**) effect of vWF silencing on NO production (green) in resting, AngII-treated cells, and cells treated with AngII and Bradykinin. (**E**) Green fluorescence intensity was quantified as Arbitrary Units by confocal microscopy (see methods sections). SiRNA NT: non-targeting siRNA; siRNA vWF: anti-vWF siRNA; AngII: angiotensin II. *p < 0.05 vs siRNA-NT at rest; ^#^p < 0.05 vs siRNA-vWF under AngII + Bradykinin. All measurements are mean ± SD, n = 3 independent experiments performed in triplicate.

An interaction between ET-1 and NO systems under the influence of Ang II has been described^13^. The chronic exposure to Ang II reduced the levels of Ser1177-phospho-eNOS/eNOS ratio (Fig. 3A,C) regardless of an increase of the total eNOS expression (Fig. 3B) in wild-type PAOECs. Conversely, the treatment with siRNA-vWF reduced eNOS protein expression (Fig. 3B) without producing any significant changes in Ser1177-phospho-eNOS/eNOS ratio both in normal conditions and under chronic exposure to Ang II (Fig. 3C).

In order to better analysis the ability of PAOECs in producing NO, we performed DAF-FM diacetate staining of cells in each experimental condition at rest and following bradykinin stimulation. As shown in Fig. 3D, bradykinin-induced NO production was significantly increased in resting wild-type and vWF-knockdown cells. Even though NO production was significantly reduced in wild-type cells chronically exposed to Ang II, the response to bradykinin stimulation of vWF-knockdown cells was preserved in presence of Ang II (Fig. 3E).

### Gene silencing of endothelial vWF prevents Ang II-induced O2− production, peroxynitrite levels, NOX expression and NADPH oxidase activity

**Figure 4:**
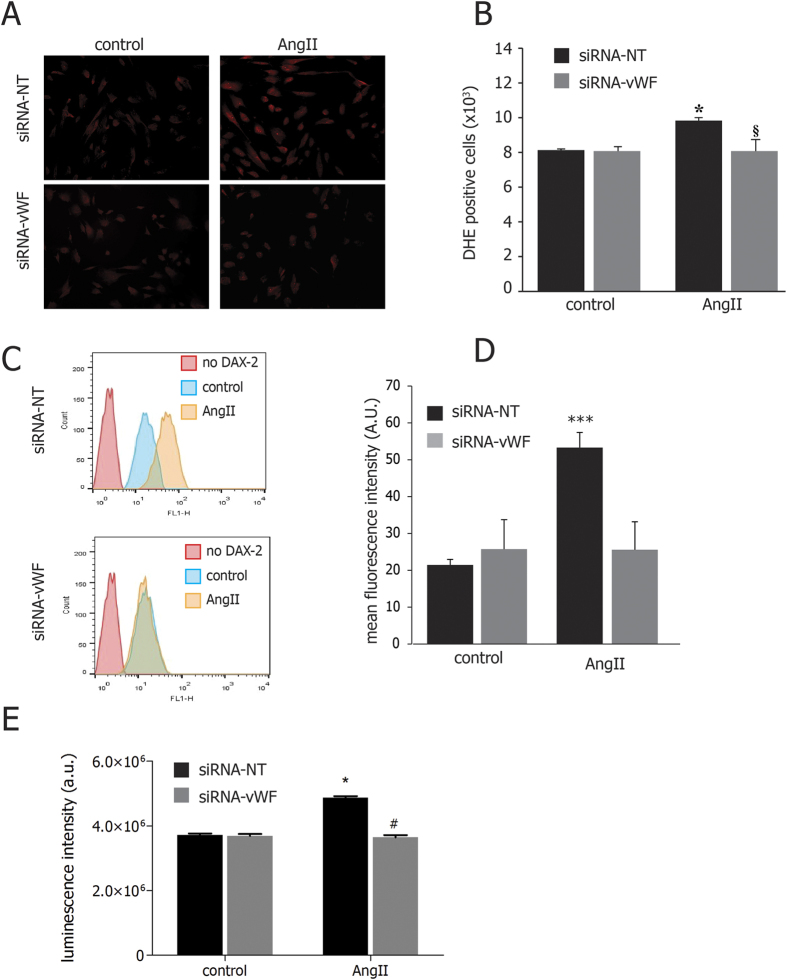
Effects of gene silencing of endothelial vWF on Ang II-induced O2− production, peroxynitrite levels and NADPH oxidase activity. (**A**) Representative images of DHE staining of wild type and vWF-knockdown cell in resting conditions and after the exposure to AngII. (**B**) vWF downregulation prevented the increase of anion superoxide generation; the results are shown as number of DHE positive cells per sample; (**C**) vWF downregulation prevented the increase in peroxynitrite levels observed in control cells after exposure to AngII. In panel **D**, results are shown as arbitrary units of fluorescence intensity; (**E**) vWF downregulation prevented the increase in NADPH activity levels observed in control cells after exposure to AngII. Results are shown as arbitrary units of luminescence intensity. siRNA NT: non-targeting siRNA; siRNA vWF: anti-vWF siRNA; AngII: angiotensin II. All measurements are mean ± SD, n = 3 independent experiments performed in triplicate. *p < 0.05 vs. control at rest; ^§^p < 0.05 vs. siRNA-NT under AngII.

As shown in Fig. 4A,B, the increase of O2−production induced by Ang II was prevented in vWF-knockdown cells. In addition, peroxynitrite levels, which were increased upon AngII treatment in control cells, remained unaltered in vWF-silenced cells (Fig. 4C,D). Similarly, the rise of Ang II-induced NADPH oxidase (NOX) activity detected in wild-type cells was prevented in vWF-knockdown cells exposed to Ang II (Fig. 4E).

### Gene silencing of endothelial vWF prevents PMA-induced O2− production, peroxynitrite levels, NOX and ET-1 expression

In order to better clarify the role of NOX in regulating the ET-1 expression in stressed silenced cells, we long-term incubated wild-type and vWF knockdown PAOECs with PMA, which increases NOX4-dependent O2− generation^29^.

**Figure 5:**
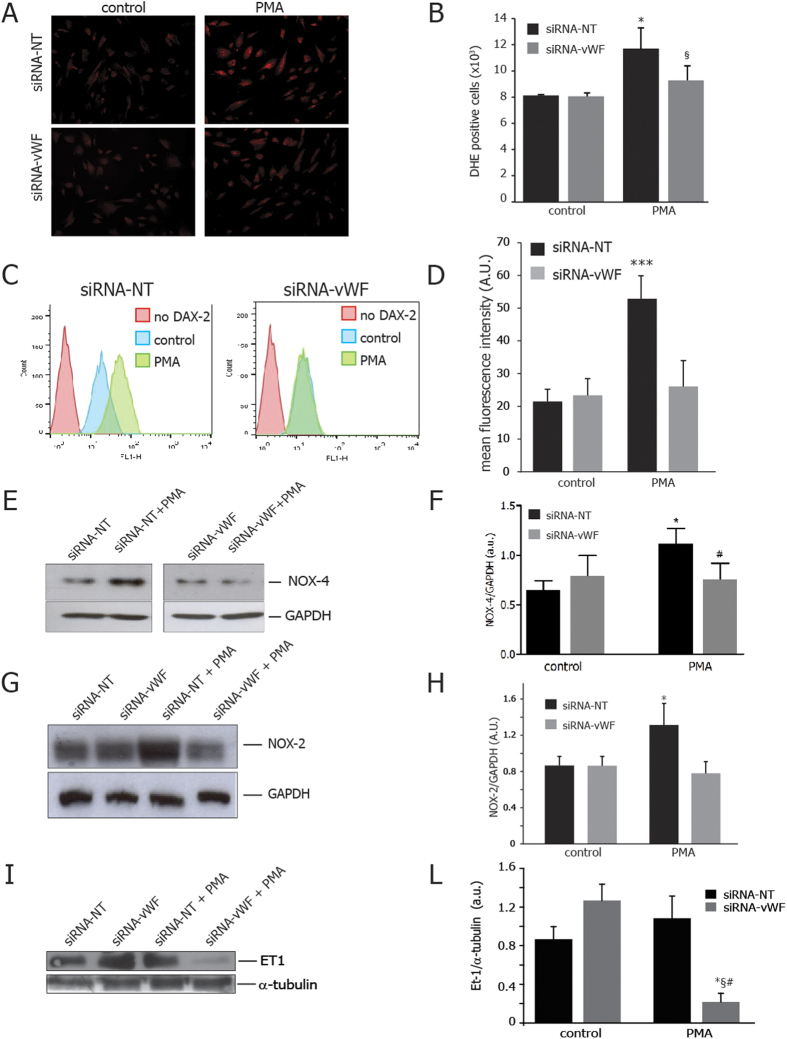
Effects of gene silencing of endothelial vWF during NOX activation. (**A**) Representative images of DHE (dihydroethidium) staining of wild type and vWF-knockdown cell in resting conditions and after the exposure to PMA. vWF downregulation prevented the increase of anion superoxide generation; in panel **B**, the results are shown as number of DHE positive cells per sample; (**C**) vWF downregulation prevented the increase in peroxynitrite levels observed in control cells after exposure to PMA; in panel **D**, results are shown as arbitrary units of fluorescence intensity. (**E**) vWF downregulation prevented the increase in NOX-4 subunit expression observed in control cells after exposure to PMA. In panel F, levels of NOX-4 are expressed as arbitrary units of NOX-4 (70 kDa, MW)/GAPDH (37 kDa, MW) ratio. (**G**) vWF downregulation prevented the increase in NOX-2 subunit expression observed in control cells after exposure to PMA. In panel H, levels of NOX-2 are expressed as arbitrary units of NOX-2 (67 kDa, MW)/GAPDH (37 kDa, MW) ratio. (**I**) vWF silencing downregulates ET-1 expression after exposure to PMA. ET-1 levels do not increase in response to PMA in vWF silenced cells. In panel L, levels of ET-1 are expressed as arbitrary units of ET-1 (24 kDa, MW)/alpha-Tubulin (50 kDa, MW) ratio. experiments performed in triplicate. *p < 0.05 vs. siRNA-NT at rest; ^#^p < 0.05 vs. siRNA-vWF at rest; ^§^p < 0.01 vs. siRNA-NT under PMA.

As shown in Fig. 5A,B, the increase of O2− generation induced by PMA was prevented in vWF knockdown cells. Similarly, peroxynitrite levels, which were increased upon PMA treatment in control cells, remained unaltered in vWF-silenced cells (Fig. 5C,D).

PMA treatment caused an upregulation of both NOX2 (Fig. 5C,D) and NOX4 (Fig. 5E,F) expression, but such effects were prevented in vWF knockdown cells.

NOX4 expression was also detected by immunofluorescence (Suppl. File: Fig. 3). Interestingly, ET-1 expression was not increased in vWF knockdown cells during chronic treatment with PMA (Fig. 5G,H). No changes were revealed in PKC expression, cell viability and growth after PMA treatment (Suppl. File: Fig. 4).

### Gene silencing of endothelial vWF prevents SAPK/JNK activation

To further elucidate how vWF knockdown prevent the PMA-induced increase in O2− generation and NOX4 expression, we investigated the phosphorylation levels of SAPK-JNK, a known regulator of NOX4 activation pathway.

**Figure 6:**
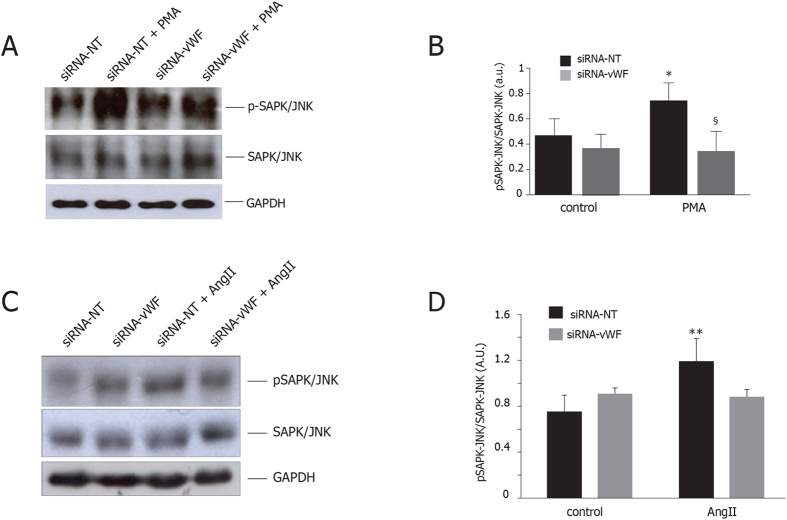
Effects of gene silencing of endothelial vWF on SAPK/JNK activation. (**A**) vWF downregulation prevented the increase in SAPK-JNK activation observed in control cells after exposure to PMA; SAPK-JNK, pSAPK-JNK (50 kDa, MW), GAPDH (37 kDa, MW). (**B**) SAPK-JNK activation is expressed as arbitrary units of pSAPK-JNK/SAPK-JNK ratio. All measurements are mean ± SD, n = 3 independent experiments performed in triplicate. *p < 0.05 vs. siRNA-NT at rest; ^#^p < 0.05 vs. siRNA-vWF at rest; ^§^p < 0.01 vs. siRNA-NT under PMA. (**C**) vWF downregulation prevented the increase in SAPK-JNK activation observed in control cells after exposure to AngII; SAPK-JNK, pSAPK-JNK (50 kDa, MW), GAPDH (37 kDa, MW). (**D**) SAPK-JNK activation is expressed as arbitrary units of pSAPK-JNK/SAPK-JNK (50 kDa, MW) ratio. All measurements are mean ± SD, n = 3 independent experiments performed in triplicate. **p < 0.05 vs. siRNA-NT at rest. siRNA NT: non-targeting siRNA; siRNA vWF: anti-vWF smart pool siRNA; AngII: angiotensin II; PMA: phorbol 12-myristate 13-acetate; SAPK/JNK: stress-activated kinase/c-Jun N-terminal kinase.

As show in Fig. 6A,B, PMA treatment significantly increased the pSAPK-JNK/SAPK-JNK ratio in control cells, while this effect was prevented by vWF silencing.

The activation of signal pathway investigated in PMA-treated cells was confirmed in PAOECs chronically exposed to AngII. In fact, vWF silencing prevented the rise of pSAPK-JNK/SAPK-JNK ratio induced by AngII (Fig. 6C,D). Finally, NOX2 and NOX4 protein levels of vWF-knockdown cells were not increased in presence of Ang II (Suppl. File: Fig. 5).

## Discussion

In the present study, we have demonstrated that endothelial vWF plays a key role in modulating ET-1 expression under stress. The rise in ET-1 levels induced by chronic exposure to Ang II was safely abolished in vWF-knockdown PAOECs. In fact, siRNA-based downregulation of endothelial vWF did not impair viability, growth and function of porcine aortic endothelial cells. Our data revealed, for the first time, that vWF plays a hitherto unsuspected pivotal role in attenuating the endothelial response to Ang II, which leads to endothelial dysfunction^11^.

It has long been known that Ang II impairs eNOS activity^10^ and simultaneously increases endothelial levels of ET-1^9^, O2−30 and vWF^31^, leading to the development of vascular dysfunction^12^ and microthrombotic coronary occlusions^19^.

Although high levels of circulating Ang II, ET-1 and vWF in the human coronary sinus reflect the occurrence of coronary endothelial injury^32^, vWF blockade improves endothelial function in coronary patients^25^ by a so far unknown mechanism.

We therefore hypothesized that downregulation of endothelial vWF expression could protect endothelial cells against Ang II exposure through inhibition of ET-1 expression.

Our hypothesis is also supported by evidence that gene silencing of endothelial vWF enhances vasculogenesis^33^, which is one of the main adaptive response of mature endothelial cells to Ang II-induced oxidative damage^34^. Accordingly, we found that an effective downregulation of vWF protein expression (−65 ± 2%), consistent with real time RT-PCR analysis, does not impair the viability and growth of our cells even during chronic exposure to Ang II.

Although the Ang II-induced upregulation of vWF is prevented in silenced cells, the vWF protein levels in stressed knockdown cells are similar to resting wild-type cells. Since Ang II does not affect vWF release from endothelial cells^35^, it is conceivable that the higher vWF levels in knockdown cells during stress rather than at rest depend on the reduction in vWF degradation induced by Ang II^36^. Our suggestion is well supported by previous clinical observations showing how the activity of ADAMTS13, a zinc containing metalloprotease that cleaves vWF, is reduced in malignant hypertension^37^ where endothelial cells are chronically exposed to high levels of Ang II^38^.

Resting ET-1 production in vWF-knockdown cells is similar to wild-type cells, but Ang II-induced ET-1 expression is abolished in PAOECs transfected with siRNA anti-vWF. It is conceivable that Ang II might synergize with ET-1 gene downregulation. In fact, the trigger of Ang II receptors activates PKC^39^, which even induces neutral endopeptidase 24.11, a membrane-bound metallopeptidase that cleaves prepro-ET1^40^. The cleavage of prepro-ET1 may enhance the deficiency of *de novo* synthesis of ET-1 by reducing its levels below baseline. Our data candidate vWF as a new upstream modulator of endothelial response to Ang II as ATR1 mRNA levels are similar in stressed wild-type and knockdown cells.

Since NO is important for endothelial cells viability and may counteract ET-1 expression^15^, we first have evaluated eNOS activity in each experimental condition. Despite the relative eNOS expression is significantly reduced in knockdown cells, the levels of phospho-Ser1177eNOS/eNOS ratio, a major hallmark of eNOS activity^41^, is similar in both cell types even during Ang II treatment. It is reasonable that compensatory mechanisms may contribute to the maintenance of eNOS enzymatic activity. In order to support our hypothesis, we have also measured the levels of NO produced in both wild-type and vWF knockdown cells after stimulation by bradykinin, a peptide that induces NO production^28^. We found that NO response to bradykinin in vWF-knockdown cells is preserved in each experimental condition. Our results strongly support the hypothesis that eNOS activity is preserved in vWF knockdown cells and plays more relevant role in endothelial NO production than total eNOS protein levels, as previously demonstrated by Iwakiri and colleagues^42^. It is noteworthy that endothelial vWF plays a key role in preventing Ang II-induced ET-1 expression independently of changes in NO production. We cannot exclude that other mechanisms are involved in regulating vWF/ET-1 pathway under stress.

It is well known that Ang II increases the levels of endothelial O2−36, which is a strong activator of the ET-1 expression^36^,^43^. In our study, Ang II-induced O2− production is increased in wild-type but not in knockdown cells. To best of our knowledge, NADPH oxidase (NOX) is the main modulator of O2− rate of production in endothelial cells exposed to Ang II^36^. Surprisingly, we found that the increase of Ang II-induced NOX activity is prevented in vWF-knockdown cells.

In order to better assess the positive ratio between O2− and NO production in silenced cells, we measured intracellular levels of peroxynitrite, a highly reactive intermediate known to induce serious oxidative damage at higher concentrations^44^. The chronic exposure to AngII or PMA has stimulated a marked increase of peroxynitrite levels in wild-type cells, which is prevented in vWF-knockdown cells.

Unraveling the mechanisms underlying vWF-dependent modulation of NOX activity and O2− production, we have chronically treated additional wild type and knockdown cells with PMA, which directly activates the expression of NOX subunits^30^ and simultaneously reduces eNOS activity through dephosphorylation of Ser-1177-eNOS^45^. Therefore, PMA is an appropriate *stimulus control* in order to investigate the role of vWF on NOX activity during simultaneous exposure to eNOS deficiency and superoxide overload.

NOX-2 and NOX-4 are the most expressed NOX isoforms in endothelial cells^46^ and the main source of superoxide in Ang II-stimulated cells^47^,^48^,^49^. Compared to wild type cells, long-term PMA treatment did not increase the O2− levels in vWF-knockdown cells showing lower levels of eNOS activity and ET-1 protein expression, as well as lower levels of peroxynitrite.

Interestingly, we found similar levels of NOX2 and NOX4 expression in vWF knockdown cells exposed to PMA or AngII. In fact, the lack of NOX2 and NOX4 expression is proportionally related to deficiency of NADPH oxidase activity in endothelial cells^50^. Taken together, our results highlight that endothelial vWF plays a key role in attenuating NADPH oxidase activation through the modulation of NOX2 and NOX4 expression, even if NOX2 is not always involved in increasing cardiac oxidative/nitrosative stress^51^. Although, vWF downregulation blocks the superoxide-induced ET-1 expression in stressed endothelial cells. How vWF deals with distinct NOX isoforms remains poorly understood.

It has already demonstrated that vWF is essential to activate c-Jun amino-terminal kinase (SAP/JNK)^52^, which is required for activation of c-jun^53^, a well-known activator of NOX4 promoter region^50^,^54^. In our study, we have demonstrated that SAPK/JNK is not phosphorylated in stressed vWF-knockdown cells exhibiting lower levels of O2− and ET-1.

In addition, previous study has reported that NOX2 expression is related to activation of JNK by phosphorylation^55^. Our results suggest that inhibition of SAPK-JNK phosphorylation may interfere with endothelial expression of NOX4 and NOX2 as well.

**Figure 7:**
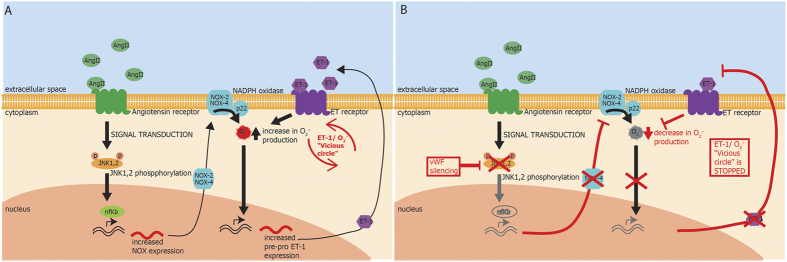
Scheme of the proposed mechanism of vWF-mediated regulation of ET-1 expression. (**A**) Scheme of mechanism leading to the onset of ET-1/O2− “vicious circle” in wild type endothelial cells chronically exposed to AngII. (**B**) Scheme of proposed mechanism of gene silencing of endothelial vWF in disabling ET-1/O2− “vicious circle” through inhibition of SAPK/JNK activation by Ang II. AngII: angiotensin II; O2−: superoxide anion; ET-1 endothelin 1; vWF: von Willebrand factor; SAPK/JNK: stress-activated kinase/c-Jun N-terminal kinase.

Therefore, by means of blocking superoxide-driven SAPK-JNK activation, vWF gene silencing may prevent the onset of the self-perpetuating loop of vascular dysfunction due to enhanced NOX activation by ET-1^14^ during continuous exposure to Ang II (the proposed mechanism is summarized in Fig. 7).

### Limitations of the study

Even if PAOECs are a well-established *in vitro* model to investigate cellular response to vascular insult^56^, clinically relevant *in vivo* studies are mandatory to confirm our findings. However, our results support previous studies demonstrating that sudden reduction in coronary blood flow following acute injury is prevented in von Willebrand’s Disease (vWD) pigs^57^ and the endothelium of vWD pigs is protected from developing endothelial lesions during intake of high-cholesterol diet^58^, which is a well-established source of oxidative/nitrative stress^51^.

## Conclusions

This is the first study to show *in vitro* data demonstrating that gene silencing of endothelial vWF prevents Ang II-induced ET-1 upregulation without interfering with the ability of porcine endothelial cells to produce NO and to sense Ang II. Lower ET-1 levels in stressed knockdown PAOECs is due to lower responsiveness of NOX to Ang II, which decreases the endogenous production of O2−, a major trigger of endogenous ET-1 synthesis. NOX unresponsiveness to common activators is driven by the blocking of SAPK/JNK pathway due to lack of vWF, which normally mediates NOX4 expression and increases superoxide production.

Our findings reveal a hitherto unsuspected role of vWF in the modulation of vascular tone and will be helpful to design a novel therapeutic approach in the clinical management of prevention of Ang II-induced vascular endothelial dysfunction.

## Methods

### Reagents

Ang II (Sigma Chemical Co, MO, USA) was dissolved in sterile water and stored at −20 °C. Phorbol 12-myristate 13-acetate (PMA) (Sigma Chemical Co, MO, USA) was dissolved in dimethyl-sulfoxide (DMSO) at a concentration of 62 mg/ml and stored at −20 °C until needed for experiments. Bradykinin (Sigma Chemical Co, MO, USA), a well-known trigger of NO production^28^, was dissolved in water at a concentration of 1 mM and stored at −20 °C until further use. DAF-FM (4-Amino-5-Methylamino-2′,7′-Difluorofluorescein) diacetate (Sigma Chemical Co, MO, USA) was dissolved in DMSO at a concentration of 5 mM and stored at −20 °C in the dark until further use.

### Endothelial cell culture

Primary porcine aortic endothelial cells (PAOECs) were purchased from Cell Applications, Inc. (San Diego, CA, USA) and cultured in specific porcine endothelial cell growth medium (PECGM) (Cell Applications, Inc., CA, USA) supplemented with 5% fetal bovine serum in humidified air with 5% CO_2_ at 37 °C. Cell were seeded initially into 75 cm^2^ tissue culture flasks and grown until they reached confluence, usually 5–7 days. Complete medium was replaced every 2 days. All experiments were performed in cells between passages 2 and 8.

### RNA interference

RNA interference was carried out using siRNAs that specifically target vWF (10 or 25 nM final concentration) (siGENOME SMARTpool M-009754-01-0005 human vWF, Dharmacon-Fisher Scientific, Italy) (Suppl. File:Table 1). Alternatively, control cells were transfected with non- targeting scrambled siRNA (siRNA-NT; silencer negative control siRNA; Shanghai GenePharma Co,Ltd., China):-5′-UUC UCC GAA CGU GUC ACG UTT-3′; Cell cultures between 80% and 90% confluence were transiently transfected with selected siRNAs using INTERFERin (Polyplus -transfection, France) following manufacturer’s instructions. After 5 h of cell incubation with transfection cocktail, PAOECs were incubated in fresh growth medium for 48 h. Western blot and Real-Time PCR assay were used to assess the efficiency of siRNAs transfection.

### Experimental protocol

At 24 h of incubation after siRNA-based transfection, PAOECs were washed twice with phosphate buffered solution (PBS) and treated for the following 24 h with vehicle (sterile water) or AngII (100 nM). The dose of Ang II was selected in accord to previous studies^59^. Cells transfected with siRNA-NT (wild-type cells) were treated with similar dose of AngII. In order to evaluate the ability of endothelial cells to produce nitric oxide (NO), wild-type and vWF-knockdown PAOECs were transiently stimulated with bradykinin (30 nM for 10 min), as previously described^28^.

In additional experiments, confluent PAOECs transfected with siRNA-NT or siRNA-vWF were long-term treated with PMA (5 nM/48 h), a potent activator of NOX4 gene expression, which increases endogenous O2− production^29^. The final concentration of DMSO (PMA vehicle) in the culture medium was less than 0.1%.

### Western blotting assay

Cell pellets were lysed in RIPA buffer containing protease and phosphatase inhibitors (Thermo Fisher Scientific, MA, US). Homogenates were then centrifuged at 10000 rpm for 10 min at 4 °C to remove nuclei and cell debris. All samples were stored at −80 °C until use. Total protein concentration was assessed using Pierce BCA Protein Assay Kit (Thermo Fisher Scientific, MA, US). Equal amounts of protein were resolved on 12% and 15% SDS-polyacrylamide gel and transferred to polyvinylidene difluoride (PVDF) membrane. Membranes were blocked with 5% milk in TBS/Tween (0, 01%) at room temperature for 1 hour, and then incubated with primary antibodies at a predetermined concentration overnight at 4 °C. Primary antibodies were used to detect vWF (1:1000; Abcam Inc., Cambridge, UK), prepro-ET-1 (1:500; Abcam Inc., Cambridge, UK), eNOS (1:1000; BD Transduction Laboratories, CA, USA), phospho-Ser1177-eNOS (1:1000; Cell signaling Technology, USA), alpha-tubulin (1:1000, Thermo Fisher Scientific, MA, US), GAPDH (Thermo Fisher Scientific, MA, US), NOX-2 (1:1000, Abcam Inc., Cambridge, UK), NOX4 (1:1000; Novus Biologicals, Littleton CO), SAPK/JNK (1:1000; Cell Signaling Technology, USA) and Phospho-SAPK/JNK (Thr183/Tyr185) (1:1000; Cell Signaling Technology, USA) .

Prepro-ET1 is a proper hallmark of ET-1 gene transcription which is the main regulator of its expression. The ratio of phospho-Ser1177eNOS (p-eNOS) and total eNOS, a hallmark of eNOS activity^41^, was determined as previously described^60^. After incubation with the abovementioned antibodies, and rinsing with TBS/Tween 20 (0.01%) 3 times for 10 min, the membranes were then incubated with appropriate horseradish peroxidase-conjugated (HRP-conjugated) anti-rabbit or anti-mouse secondary antibodies (Abcam Inc., Cambridge, UK) for 1 hour at room temperature. Specific protein bands were detected using Pierce ECL Western blotting substrate (Thermo Fisher Scientific, MA, US). Protein bands were quantified using ImageJ software.

### NO production fluorescent assay

DAF-FM diacetate staining for the determination of intracellular nitric oxide bioavailability in PAOEC cells was performed as described elsewhere^61^. Briefly, PAOECs were seeded on WillCo-dish^®^ glass bottom dishes (Willco Wells, Amsterdam, NL). Wild-type (siRNA-NT) and siRNA-vWF transfected cells were treated for 24 h with 100 nM AngII or saline solution (Vehicle) and then stimulated with bradykinin, as abovementioned. Unstimulated cells served as the negative control. In order to assess NO production, cells were loaded with 10 uM of the NO-sensitive fluorescence probe DAF-FM diacetate (Sigma Chemical Co, MO, USA) in the dark at 37 °C for 30 min; cells were then washed and incubated for further 15 min in fresh medium at 37 °C to allow complete de-esterification of the probe. At the end of an experiment, fluorescence was detected within 10 fields for each condition and analyzed with a Leica DM IRE 2 confocal microscope with a setting of 40x magnification. Relative fluorescence intensity per cell was quantified using ImageJ software. The experiments were performed in triplicate.

### Superoxide Anion assay

Endothelial superoxide generation was determined by staining PAOECs with fluorescent-labeled dihydroethidium (DHE; Invitrogen, CA, USA). DHE is cell permeable and reacts with superoxide to form ethidium, which in turn intercalates with DNA and produces nuclear fluorescence.

Cells were treated with 5 mol/L DHE in phosphate buffered saline for 30 min at 37 °C as previously described^61^,^62^. Positive nuclear DHE staining is an indicator of superoxide generation in cells. In general, 5 to 6 images of cells were captured randomly by a Leica DM2500 fluorescence microscope with a setting of 40x magnification. The total number of nuclear DHE positive cells in all images was counted by using threshold ImageJ algorithms to discriminate the features of interest from background. Individual images were converted to grayscale by RGB splitting, and the red channel was used for DHE staining. Images were thresholded to separate positively stained areas, which were then sujected to cell counting. The colour threshold was set the same for each image.

### NADP/NADPH activity luminescent assay

The NADP/NADPH-Glo™ Assay (Promega Italia S.R.L., Milan, Italy) was used to detect total oxidized and reduced nicotinamide adenine dinucleotide phosphates in PAOECs. Bioluminescent detection assay was performed following manufacturer’s instruction. Briefly, PAOECs were seeded at the same concentration (10^4^ per well) in triplicate in 24 well plates for 6 h prior to the addition of an equal volume (50 μl) of the NADP/NADPH-Glo TM detection reagent to each cell suspension (50 μl). The luminescence was read after incubation with the detection reagent for 60 min at room temperature on a GloMax luminometer (Promega Italia S.R.L., Milan, Italy). All readings were performed in triplicate, and the graph shows the result of three independent experiments.

### Fluorimetric Intracellular Peroxynitrite Assay

Intracellular levels of peroxynitrite were determined using Cell Meter Fluorimetric Intracellular Peroxynitrite Assay kit (AAT Bioquest Inc., Sunnyvale, CA,US), according to manufactorer’s instructions.

DAX-J2™ PON Green is developed as an excellent fluorescent probe, which can specifically react with intercellular ONOO^−^ to generate a bright green fluorescent product. Briefly, after the silencing protocol and treatment with AngII or PMA, cells were washed, tryspinized, resuspended in fresh complete medium, and incubated with DAX-J2 PON probe, for 1 h at 37 °C. Finally, cell fluorescence was quantified by flow cytometry using a FACScalibur (BD biosciences, San Jose, CA, US).

### Statistical Analysis

The statistical analysis was performed using GraphPad Prism ver. 5. All results are presented as mean ± SD. All experiments were performed in triplicate. Statistical comparisons were made by analysis of variance (ANOVA) and Bonferroni test was used as the post-hoc test. p < 0.05 was considered statistically significant.

## Additional Information

**How to cite this article**: Dushpanova, A. *et al*. Gene silencing of endothelial von Willebrand Factor attenuates angiotensin II-induced endothelin-1 expression in porcine aortic endothelial cells. *Sci. Rep.* **6**, 30048; doi: 10.1038/srep30048 (2016).

## Supplementary Material

Supplementary Information
