# Supplementary material for: Gene silencing of endothelial von Willebrand Factor attenuates angiotensin II-induced endothelin-1 expression in porcine aortic endothelial cells
Source: Sci Rep. 2016 Jul 22;6:30048. doi: 10.1038/srep30048 (PMC4957110; doi:10.1038/srep30048)
Supplement: Supplementary Information [file srep30048-s1.pdf]

Supplementary File

Gene silencing of endothelial von Willebrand Factor attenuates angiotensin II-induced endothelin-1 expression in porcine aortic endothelial cells

Dushpanova Anar[1#], Agostini Silvia[1#], Ciofini Enrica[1,2], Cabiati Manuela[3], Casieri Valentina[1], Matteucci Marco[1], Del Ry Silvia[3], Clerico Aldo[1, 2], Berti Sergio[2], Lionetti Vincenzo[1,2*]

Supplementary methods

MTT assay

Cell viability and growth were determined by MTT assay (Sigma Chemical Co, MO, USA) according to the manufacturer's instructions. Briefly, cells were seeded into 96-well plates at a density of $6x10^3$cells/150μl/well.

After 24h of culture, wild-type and vWF knockdown cells were long-term treated with Ang II (24h) or PMA (48h). At the end of each treatment, medium was removed; the cells were washed with PBS and incubated with 3-(4,5 dimethylthiazol-2-yl)-2,5-diphenyl tetrazoliumbromide, an indicator of metabolic activity. After 4h, MTT solution was removed and MTT Solvent (isopropanol) was added. Plates were gently stirred in a gyratory shaker for 10 minutes to enhance complexes dissolution and read within 30min from MTT Solvent addition and absorbance (O.D.) was measured.

In order to measure the cell viability, we used the following equation:

viable cells (%)=(O.D. of treated sample/O.D. of untreated sample)×100 [1]

In order to measure the cell growth, we used the following equation:

cell growth= (O.D. 540nm-O.D. 660nm) [2].

The analysis was performed in three independent experiments performed in triplicate.

Real-Time PCR assay

Total RNA was extracted using an RNAesy kit (Qiagen, Hilden, Germany) and retrotranscribed with Reverse iScript cDNA Synthesis Kit (Bio-Rad Laboratories Inc., Hercules, CA, USA) according to the manufacturer's instructions. The trascriptomic profile of endothelial vWF and angiotensin II receptor type 1 (ATR1) was assessed by Real-Time PCR assay. The reactions were performed in duplicate in the Bio-Rad C1000™ thermal cycler (CFX-96 Real-Time PCR detection systems, Bio-Rad Laboratories Inc., Hercules, CA, USA) as previously described[3,4]. EvaGreen, a third-generation fluorophore, was used to monitor cDNA amplification (SsoFAST EvaGreen Supermix, Bio-Rad Laboratories Inc., Hercules, CA, USA). PCR was performed in a volume of 20μl per reaction; to minimize the influence of PCR inhibitors in Real-Time applications, all cDNA samples were diluted 1:5 and 0.2μM of each primer (Eurofins Genomics, Germany), 1X SsoFAST EvaGreen SuperMix (Bio-Rad Laboratories S.r.l., Milan, Italy) and sterile water were added.

Amplification protocol started with 98°C for 30s followed by 40 cycles at 95°C for 5s and 60°C for 30s. To assess the product specificity, melting curve analysis systematically checked amplicons. Melting curves were generated from 65°C to 95°C with increments of 0.5°C/cycle. The primer pairs specific for both target and reference genes (Suppl. Data: Table 2) were designed with Primer Express Version 2.0 (Applied Biosystems); whenever possible, intron-spanning primers were selected to avoid amplification of genomic DNA.

A standard curve obtained by scalar dilution of a cDNA pool (1:5, 1:25, 1:125, 1:625) was generated to verify PCR efficiency and a linear standard curve, R^2, greater than $|0.990|$ was generated, as reported in Supplemental Figure 1.

In an effort to provide greater transparency of our results between research laboratories, this study was carried out to conform to the *Minimum Information for publication of*

Quantitative Real-Time PCR Experiments (MIQE)[5]. A MIQE checklist was listed in Supplemental Table 2.

To verify vWF presence in samples, the amplified products of both vWF and one of the reference genes analyzed (TOP2B, Topoisomerase II beta) were then electrophoresed on 1.5% (w/v) agarose gel, making parallel with a DNA Ladder both 50 than 100 bp (Promega Corporation, USA), stained with Gel Star Stain (Lonza, Switzerland, CH) and displayed on an ultraviolet transilluminator at 260nm (Benchtop UV transilluminator, UVP, Euroclone).

GeNorm software was used to define the most stably expressed gene set, as previously described[6]. Two stably expressed reference genes were selected among ten of the most studied in literature and the geometric mean of these was used for normalization of mRNA expression (Top2B and HPRT-1, M value< 1) (Table 2). The relative quantification was performed by ΔΔCt method using BioRad's CFX96 manager software.

Western blotting assay

Western blotting procedure was performed as described in Methods section; primary antibodies were used to detect PKC (1:1000; Millipore, MA, USA), NOX-2 (1:1000, Abcam Inc., Cambridge, UK), NOX4 (1:1000; Novus Biologicals, Littleton CO), and alpha-tubulin (1:1000, Thermo Fisher Scientific, MA, US.

Immunofluorescent detection of NOX4

PAOECs were grown on chamber slides, fixed, and incubated twice for 10 min with PBS 0.1% Triton X-100 to permeabilize plasma membranes, then extensively washed in PBS; after 1h incubation with blocking buffer (PBS 2% BSA), cells were incubated with primary antibody (anti-NOX4 antibody, 1:100) for 2h at 37°C. Cells were then washed in PBS 0.5% BSA and incubated with secondary antibody (goat anti-rabbit, Alexa-488 conjugated, 1:500, Santa Cruz Biotechnology, Inc, USA) for 1h at 37°C. Slides were mounted with Vectashield antifade mounting medium

containing DAPI (Vectorlabs, CA, US) and analyzed. All antibodies were diluted in PBS 1% BSA. In general, 5 images were acquired for each condition with a Leica DM2500 fluorescence microscope with a setting of 40X magnification. Quantification of relative fluorescence intensity per cell was performed with ImageJ software and obtained data were analyzed for statistical significance with graphPad PRISM. The experiments were performed in triplicate.

REFERENCES

1. Lai LH, Fu QH, Liu Y, et al. Piperine suppresses tumor growth and metastasis in vitro and in vivo in a 4T1 murine breast cancer model *Acta Pharmacol. Sin.* 2012; 33(4):523-30.
2. Ng KW, Leong DT, Hutmacher DW. The challenge to measure cell proliferation in two and three dimensions. *Tissue Eng.* 2005;11(1-2): 182-91.
3. Del Ry S, Cabiati M, Lionetti V, et al. Pacing-induced regional differences in adenosine receptors mRNA expression in a swine model of dilated cardiomyopathy. *PLoS One.* 2012; 7: e47011.
4. Del Ry S, Cabiati M, Martino A, et al. High concentration of C-type natriuretic peptide promotes VEGF-dependent vasculogenesis in the remodeled region of infarcted swine heart with preserved left ventricular ejection fraction. *Int. J. Cardiol.* 2013; 168(3): 2426-34.
5. Bustin SA, Benes V, Garson JA, et al. The MIQE guidelines: minimum information for publication of quantitative real-time PCR experiments. *Clin. Chem.* 2009; 55(4): 611–22.
6. Martino A, Cabiati M, Campan M, et al. Selection of reference genes for normalization of real-time PCR data in minipig heart failure model and evaluation of TNF-α mRNA expression.J. Biotechnol. 2011;153(3-4):92-9.

TABLES

	Catalog number	Target sequence (5'→3')
1	D-009754-04 vWF	GGUCACAUCUUCACAUUCA
2	D-009754-03 vWF	GAAGAGGCCUGCACUCAGU
3	D-009754-02 vWF	GGAAGACCCUGUGGACUUU
4	D-009754-01 vWF	GGACAGAUCAUGACACUGA

Table 1. Target sequences of siRNA against vWF (siGENOME SMARTpool M-009754-01-0005 human vWF)

Gene	Primer sequence (5'→3')	Amplicon length	Tm,° C	GenBank n°	Chromosome map
ATR1	**F:** CTGTCTACACTGCTATGGAATAC<br>**R:** ACACTGGCATAGAGGTTGA	94 bp	62°	XM_003132469	13: 96,933,073-96,978,416
vWF	**F:** GGAGAGTCTGATGATTGATGTAT<br>**R:** CTCACAGGTGGTCTTCTTG	100 bp	60°	AF052036	5: 67,005,340-67,078,256
TOP2B	**F:** AACTGGATGATGCTAATGATGCT<br>**R:** TGGAAAAACTCCGTATCTGTCTC	137 bp	60°	AF222921	13: 14,120,032-14,187,840
HPRT-1	**F:** CCGAGGATTTGGAAAAGGT<br>**R:** CTATTTCTGTTCAGTGCTTTGATGT	181 bp	60°	DQ178126	Scaffold GL895293.1: 24,635-58,516

Table 2. RT-PCR primer sequence of genes of interest. WF: von Willebrand Factor; TBP: TATA binding protein.

SUPPLEMENTARY FIGURE LEGENDS

Supplementary Figure 1: Gene silencing of endothelial vWF does not affect cell viability and growth.

A: effects of siRNA transfection on cell viability and growth in resting conditions; B, effects of siRNA transfection on cell viability and growth under AngII. Mock: mock treated cells; AngII: angiotensin II (100nM AngII for 24h); siRNA NT: non-targeting siRNA; siRNA vWF: anti-vWF siRNA. All measurements are mean ±SD, n =3 independent experiments performed in triplicate.

Supplementary Figure 2: Real-Time PCR validation of siRNA-mediated gene silencing of vWF in PAOEC cells.

A, upper graph: Real-Time PCR result obtained for mock (green curves), siRNA non targeting (blue curves) and siRNA vWF (orange curves) belonging to cells at rest and stress (Angiotensin II); lower graph: Melting peak, rate of change of the relative fluorescence units (RFU) with time (T) (-d(RFU)/dT) on the Y-axis vs the temperature on the X-axis (peak at the melting temperature).

B: Representative electrophoresis bands on Gel Star Stain (Lonza, Switzerland, CH) agarose gels. Upper: vWF bands in correspondance of 100 bp; lower: TBP bands in correspondance of 124 bp. Orange squares mark siRNA vWF samples. On the left: resting cells (1-3: mock; siRNA non targeting; siRNA vWf); on the right: cells under Ang II (4-6:; mock; siRNA non targeting; siRNA vWf). AngII: angiotensin II (100nM AngII for 24h). Mock: mock treated cells; siRNA NT: non-targeting siRNA; siRNA vWF: anti-vWF siRNA.

Supplementary Figure 3: Immunofluorescent detection of NOX4.

A: Immunofluorescent detection of NOX4 in wild type and vWF-knockdown cells in resting conditions and after the exposure to PMA to induce superoxide production. B: levels of NOX-4 are shown as arbitrary units of fluorescence intensity by fluorescent microscopy (see methods section).

Supplementary Figure 4: Effects of PMA treatment on cell viability , growth and PKC expression in PAOEC cells.

A: Gene silencing of endothelial vWF does not interfere with cell viability and growth of cells long-term treated with PMA (5nM/48h). B: vWF downregulation did not affect PKC protein expression in cells long-term treated with PMA. Representative western blot is shown. C: Levels of PKC are expressed as arbitrary units of PKC (80kDa, MW)/alpha-Tubulin (50kDa, MW) ratio. siRNA NT: non-targeting siRNA; siRNA vWF: anti-vWF smart pool siRNA; AngII: angiotensin II; PMA: phorbol 12-myristate 13-acetate. All measurements are mean ±SD, n =3 independent experiments performed in duplicate.

Supplementary Figure 5: Expression of NOX-2 and NOX4 following AngII treatment.

A: vWF downregulation prevented the increase in NOX-4 subunit expression observed in control cells after exposure to AngII. B: levels of NOX-4 are expressed as arbitrary units of NOX-4 (70kDa, MW)/ GAPDH (37kDa, MW) ratio. C: vWF downregulation prevented the increase in NOX-2 subunit expression and SAPK-JNK activation observed in control cells after exposure to AngII. D: levels of NOX-2 are expressed as arbitrary units of NOX-2 (67kDa, MW)/ GAPDH (37kDa, MW) ratio. siRNA NT: non-targeting siRNA; siRNA vWF: anti-vWF smart pool siRNA; AngII: angiotensin II. All measurements are mean ±SD, n =3 independent experiments performed in triplicate. * $p<0.05$ vs. control at rest; § $p<0.05$ vs. siRNA-NT under AngII.

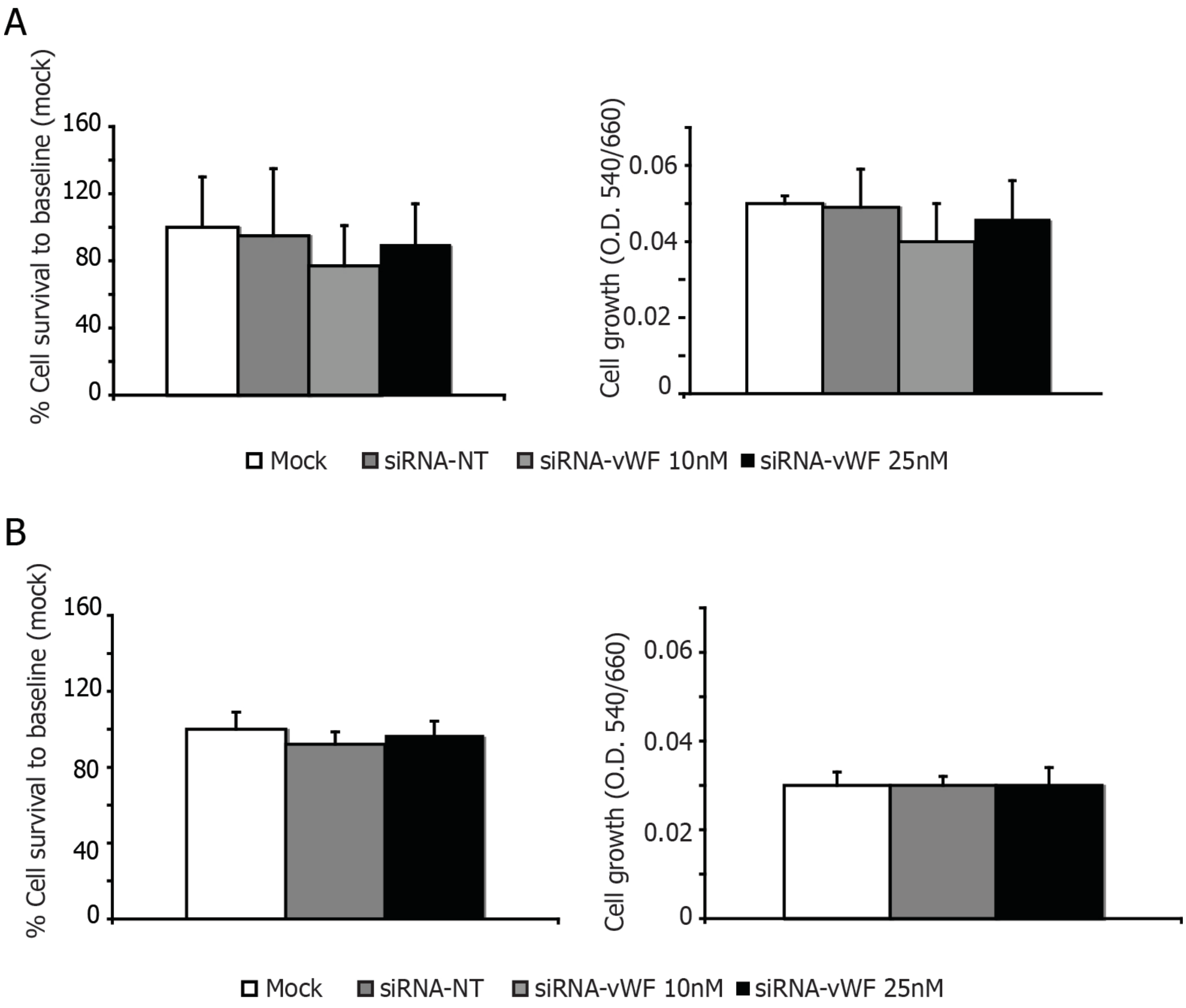

Supplementary Figure 1.

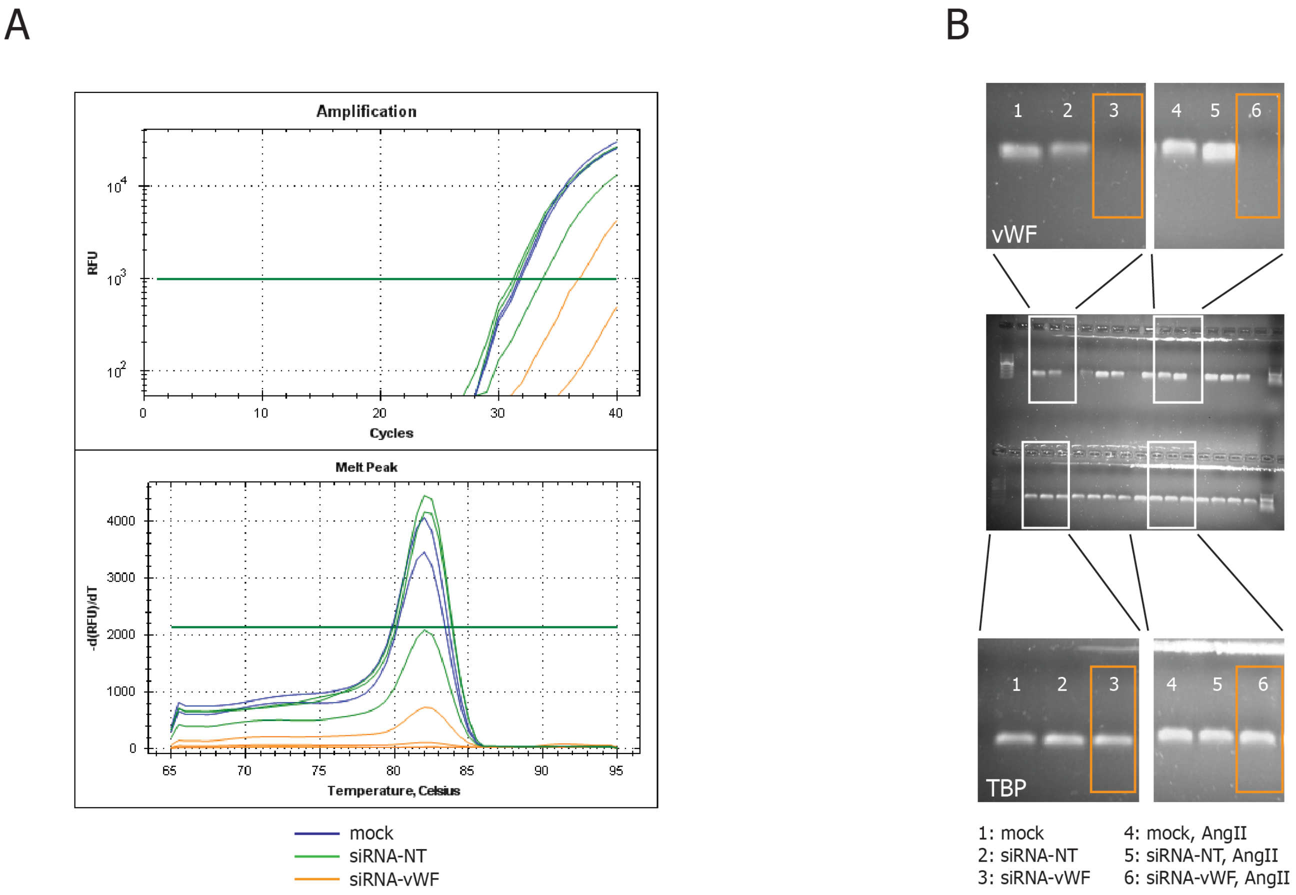

Supplementary Figure 2.

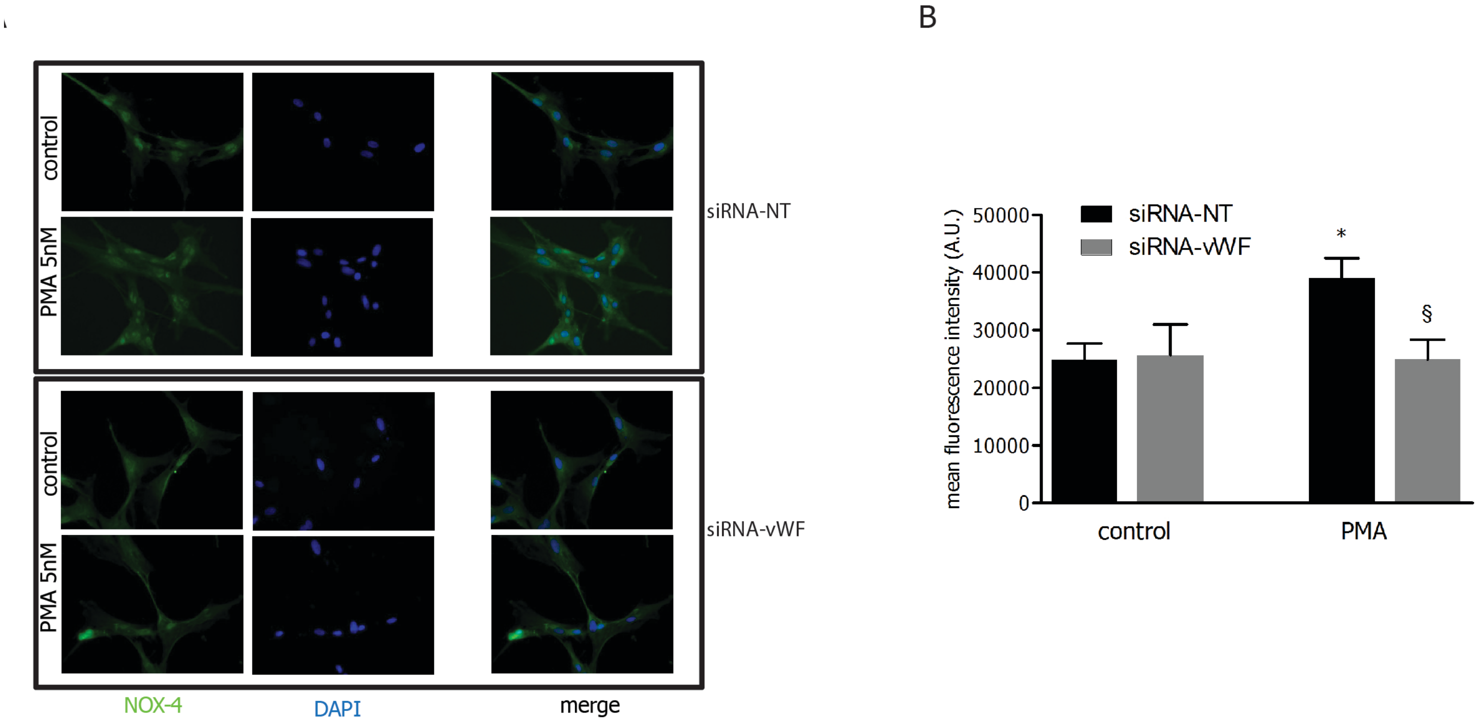

Supplementary Figure 3.

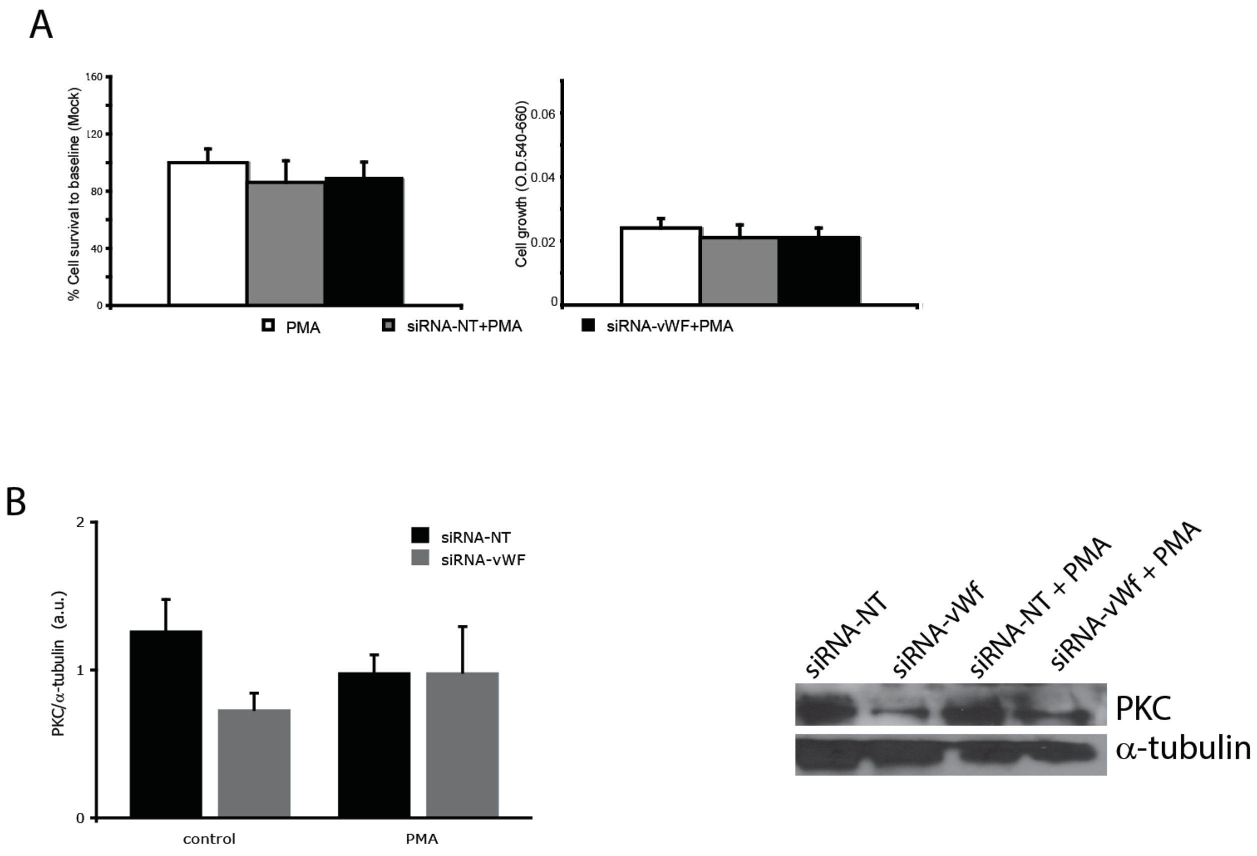

Supplementary Figure 4.

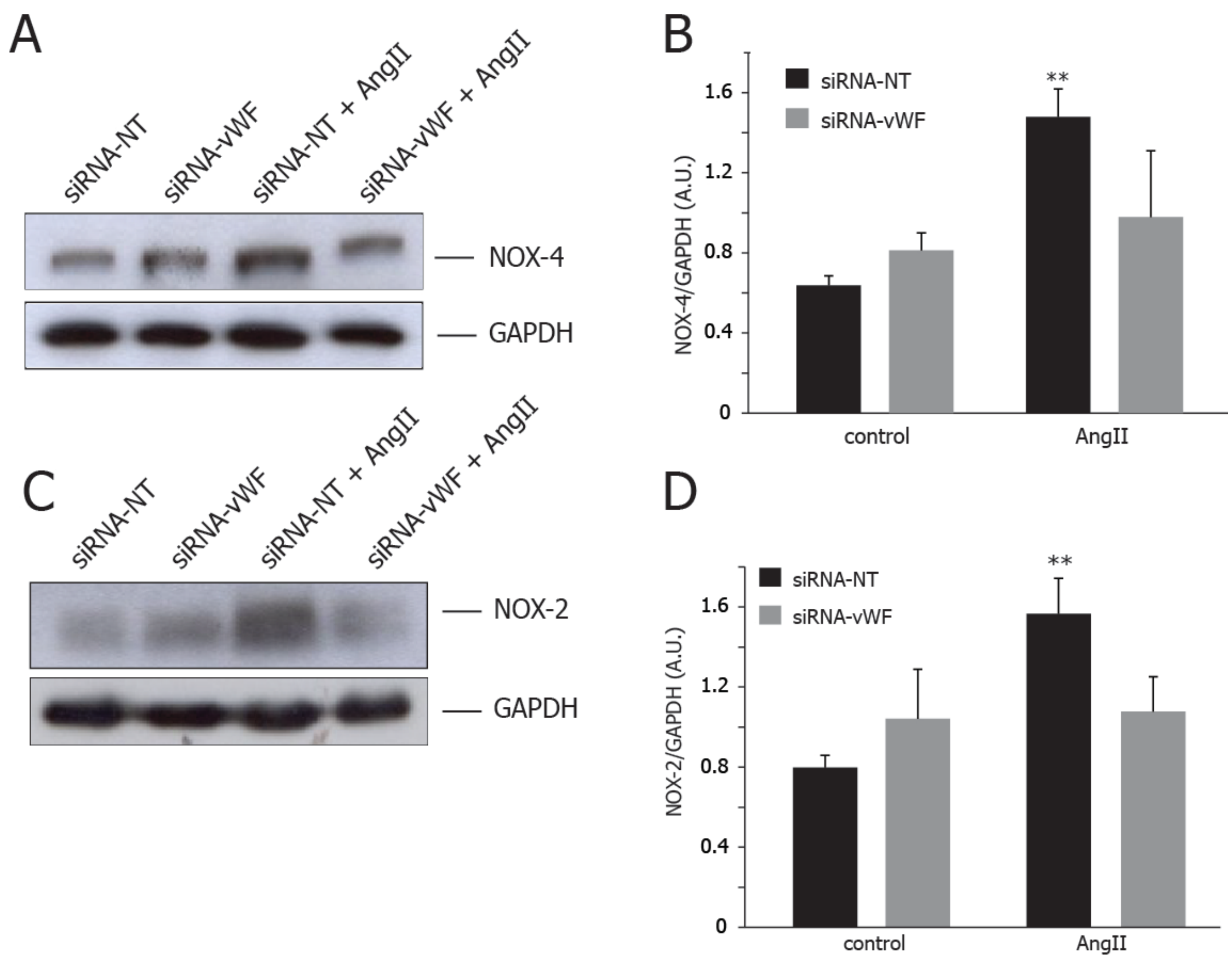

Supplementary Figure 5.
